# A Novel Immunomodulatory Hemocyanin from the Limpet *Fissurella latimarginata* Promotes Potent Anti-Tumor Activity in Melanoma

**DOI:** 10.1371/journal.pone.0087240

**Published:** 2014-01-23

**Authors:** Sergio Arancibia, Cecilia Espinoza, Fabián Salazar, Miguel Del Campo, Ricardo Tampe, Ta-Ying Zhong, Pablo De Ioannes, Bruno Moltedo, Jorge Ferreira, Ed C. Lavelle, Augusto Manubens, Alfredo E. De Ioannes, María Inés Becker

**Affiliations:** 1 Fundación Ciencia y Tecnología para el Desarrollo (FUCITED), Santiago, Chile; 2 Clinical & Molecular Pharmacology Program, Instituto de Ciencias Biomédicas (ICBM), Universidad de Chile, Santiago, Chile; 3 Adjuvant Research Group, School of Biochemistry and Immunology, Trinity Biomedical Sciences Institute, Trinity College, Dublin, Ireland; 4 Department of Research and Development, Biosonda Corporation, Santiago, Chile; Istituto Superiore di Sanità, Italy

## Abstract

Hemocyanins, the huge oxygen-transporting glycoproteins of some mollusks, are used as immunomodulatory proteins with proven anti-cancer properties. The biodiversity of hemocyanins has promoted interest in identifying new anti-cancer candidates with improved immunological properties. Hemocyanins promote Th1 responses without known side effects, which make them ideal for long-term sustained treatment of cancer. In this study, we evaluated a novel hemocyanin from the limpet/gastropod *Fissurella latimarginata* (FLH). This protein has the typical hollow, cylindrical structure of other known hemocyanins, such as the keyhole limpet hemocyanin (KLH) and the *Concholepas* hemocyanin (CCH). FLH, like the KLH isoforms, is composed of a single type of polypeptide with exposed N- and O-linked oligosaccharides. However, its immunogenicity was significantly greater than that of KLH and CCH, as FLH induced a stronger humoral immune response and had more potent anti-tumor activity, delaying tumor growth and increasing the survival of mice challenged with B16F10 melanoma cells, in prophylactic and therapeutic settings. Additionally, FLH-treated mice demonstrated increased IFN-γ production and higher numbers of tumor-infiltrating CD4^+^ lymphocytes. Furthermore, *in vitro* assays demonstrated that FLH, but not CCH or KLH, stimulated the rapid production of pro-inflammatory cytokines (IL-6, IL-12, IL-23 and TNF-α) by dendritic cells, triggering a pro-inflammatory milieu that may explain its enhanced immunological activity. Moreover, this effect was abolished when deglycosylated FLH was used, suggesting that carbohydrates play a crucial role in the innate immune recognition of this protein. Altogether, our data demonstrate that FLH possesses increased anti-tumor activity in part because it activates a more potent innate immune response in comparison to other known hemocyanins. In conclusion, FLH is a potential new marine adjuvant for immunization and possible cancer immunotherapy.

## Introduction

Adjuvants are key components of successful cancer vaccines, and biomedical research aimed at developing new adjuvants has directly improved the therapeutic efficacy of cancer vaccines [1], [2]. These substances, apart from enhancing antigen immunogenicity, are key factors in modulating the magnitude, type and profile of the immune response, thereby increasing the protective quality of a vaccine [3]. In particular, there is an increasing demand for safe and non-toxic substances that can boost cellular and humoral immune responses against cancer [4], [5]. Among potent immunostimulants, hemocyanins, the large oxygen-transporting glycoproteins found in mollusks, have been shown to trigger a strong Th1 biased cellular and humoral response against certain cancers with negligible toxic side effects, thus making them ideal for long-term repetitive treatments [6], [7].

Hemocyanins have been used clinically as non-specific immunostimulants, with beneficial outcomes, to prevent the progression of superficial bladder cancer [8], [9]. Moreover, these proteins have been fundamental tools as carriers/adjuvants for producing antibodies against tumor-associated antigens, in therapeutic vaccines against cancer [7]. Other therapeutic strategies using hemocyanins include the generation of *ex vivo* autologous tumor antigen-loaded dendritic cells (DCs) for inducing polyclonal T cell expansion [10]–[12]. Furthermore, studies have demonstrated that hemocyanins contribute to the reversal of the tolerogenic profile of DCs in cancer patients toward a more immunostimulatory profile [13]. The hemocyanin from the gastropod *Megathura crenulata*, also known as keyhole limpet hemocyanin (KLH), has mainly been used for these applications [14], [15]. However, additional hemocyanin candidates with greater immunogenicity and invariable composition are desirable. The disadvantage of KLH is related to its composition, as it comprises 2 isoforms which coexist in variable proportions in preparations of the agent [16], [17]. Accordingly, several hemocyanins from other species of gastropods have been studied [18], [19], although only the hemocyanin from *Concholepas concholepas* (CCH) has been preclinically evaluated in a murine model of superficial bladder cancer [20].

Gastropod hemocyanins are enormous glycoproteins (4 to 8 MDa) formed by a complex arrangement of 10 subunits that are self-assembled into hollow cylinders 35 nm in diameter, which are referred to as decamers [21]. This structure is easily observed by negative staining using transmission electron microscopy (TEM). Each subunit, ranging from 350 to 450 kDa, includes eight globular folded domains known as functional units (FUs), and each of them is capable of reversibly binding one oxygen molecule through a pair of copper atoms. In KLH and CCH, the decamers may associate in pairs to form huge molecules called didecamers [22]. Closer analysis has revealed that KLH and CCH differ significantly in their quaternary structures [23] and conformational stabilities [24]. Despite the structural differences between KLH and CCH, we previously demonstrated that both hemocyanins stimulate a Th1 response, suggesting an evolutionarily shared mechanism [20]. An additional feature of the hemocyanins is its carbohydrate content, which can be as high as 9% (w/w), with mannose as the most abundant oligosaccharide [14]. It has been shown that KLH induces the maturation of human DCs through the mannose receptor [13]. In addition, hemocyanins are processed very slowly by DCs, which facilitates more robust and sustained antigen presentation to CD4^+^ lymphocytes and results in augmented immunogenicity [25].

In this work, we characterize a hemocyanin isolated from the gastropod *Fissurella latimarginata*, termed FLH, which has not been previously evaluated as an immunostimulant for cancer therapy. We studied the biochemical properties of FLH, along with the immunotherapeutic value of FLH in the B16F10 mouse melanoma model, and our results demonstrate that FLH is more immunogenic than KLH and CCH and leads to decreased tumor growth and improved survival of mice. Moreover, *in vitro* stimulation of DCs showed that FLH, but not KLH or CCH, could induce the rapid production of pro-inflammatory cytokines. This effect was abolished when deglycosylated FLH was used, indicating that unique glycosylation of this protein may result in its increased immunological when compared to other hemocyanins.

## Materials and Methods

### Ethics statement

The study was carried out in strict accordance with the Guidelines for the Care and Use of Laboratory Animals (National Commission for Scientific and Technological Research, CONICYT, of Chile) The CONICYT Committee on Animal Welfare approved all animal protocols used in this study (FONDECYT 1110651). The conditions of the animals were monitored daily. Humane endpoints to reduce pain or distress were used during the survival study, considering the following criteria: physical appearance, behavior, movement, hydration, stool consistency and tumor burden. Animals were euthanized by cervical dislocation. All efforts were made to minimize suffering and the procedure was performed under anesthesia if necessary.

C57BL/6 and BALB/c mice were obtained from the Universidad de Chile (Santiago, Chile) and GrupoBios (Santiago, Chile) respectively. C3H/HeJ mice were purchased from Jackson Laboratories (Bar Harbor, Maine, USA) and were bred at Biosonda Corp. The experimental mice were housed at 22 to 24°C with a light/dark cycle of 12/12 h. Sterile water and food were available ad libitum.

### Hemocyanin sources

The hemocyanins from *Fissurella latimarginata* (FLH), *Fissurella cumingi* (FCH) and *Fissurella maxima* (FMH) were purified by Biosonda Corp., (Santiago Chile). Hemocyanins were obtained under sterile and pyrogen-free conditions and purified by precipitation at 50% saturation with ammonium sulfate, using the basic procedure of Herkowitz et al. [26], and were suspended in Tris buffer (50 mM Tris pH 7.4, mM CaCl_2_, 5 mM MgCl_2_ and 0.15 mM NaCl). The hemocyanin from *Concholepas concholepas* suspended in PBS (0.1 M sodium phosphate, 0.15 M NaCl; pH 7.2) was provided by the Biosonda Corp. Lyophilized KLH in PBS was purchased from Thermo Scientific (Waltham, Massachusetts, USA) and Calbiochem (Merck, Darmstadt, Germany). Deglycosylated FLH (Ox-FLH) was produced by sodium periodate treatment using the procedure and controls of Arancibia et al. [25]. The endotoxin contents of KLH, CCH and FLH preparations were determined using a PyroGene Recombinant Factor C Endotoxin Detection Assay kit (Lonza Group, Walkersville, USA). All chemicals were analytical grade reagents, and the solutions were prepared using water for human irrigation (Baxter Healthcare Corp., Charlotte, NC, USA) and were filtered through a 0.2-µm membrane filter (Millipore, Billerica, MA, USA).

### Cell line

Mouse B16F10 melanoma cells were grown at 37°C in a humidified atmosphere (10% CO_2_ in air) in Dulbecco's modified Eagle's medium (DMEM; Invitrogen, Carlsbad, CA, USA) supplemented with 5% heat-inactivated fetal bovine serum (FBS; HyClone, St. Louis, MO, USA), 100 U/ml penicillin, 100 µg/ml streptomycin, 2.5 µg/ml Fungizone, 1 mM sodium pyruvate and 0.1 mM non-essential amino acids (Invitrogen), as described previously [25]. The MTT (3-[4,5-dimethylthiazol-2-yl]-2,5 diphenyl tetrazolium bromide) assay (Sigma Aldrich, St. Louis, MO) according to manufacturer instructions was used to determine viability of B16F10 cells.

### Transmission electron microscopy (TEM)

The hemocyanin samples were negatively stained as described previously [23]. Briefly, aliquots of FLH (100 µg/ml) were applied to parlodion-coated copper grid. The proteins were stained with a 1–2% aqueous uranyl acetate solution and then examined and photographed at 80 kV using a Philips Tecnai-12 electron microscope at the Electron Microscopy Facility of the Pontificia Universidad Católica de Chile.

### Oligosaccharide analyses

As a first step the nature of the exposed oligosaccharides present in the hemocyanins was determined using the biotinylated lectins (Vector Labs Inc. California, USA) concanavalin A (ConA) and peanut agglutinin (PNA), as described previously [27]. Briefly, the samples (20 µg) were placed on a nitrocellulose membrane and blocked with 3% BSA in Tris-buffered saline (TBS). The membranes were then incubated with the biotinylated lectins (1 µg/ml) in TBS containing 0.3% BSA and 0.05% Tween 20, washed and incubated using 0.1 units streptavidin-ALP and then washed and developed using NBT-BCIP (Thermo Scientific). As a control, the biotinylated lectin was omitted.

The oligosaccharide profiling by MALDI-TOF-MS analysis of purified FLH and CCH was performed by GlycoSolutions Corp., (Marlborough, MA, USA). Briefly, oligosaccharides were released using non-reductive β-elimination, derivatized with 2- aminobenzoic acid, and then analyzed in negative ion mode MALDI-TOF-MS using 2,5-dihydroxybenzoic acid as the matrix. Data analysis for MALDI-TOF-MS data was performed using GlycoMod (expasy.org). A tolerance of ±3 daltons was used. The derivative mass for the 2-aminobenzoic acid was 137 g/mol.

### Electrophoretic methods

The native electrophoresis of proteins was performed using a horizontal gel chamber and 1.5% agarose gels prepared in 70 mM Tris and 261 mM boric acid pH 7.4 [27]. The proteins were visualized with Coomassie blue staining. SDS-PAGE analyses were performed using gradient separating gels (5–15%) according to the standard procedure [28]. Molecular masses were estimated based on band mobility with a calibration curve obtained from CCH and KLH as well as pre-stained markers (Invitrogen). In all of these analyses, FCH and FMH were also incorporated as controls.

### HPLC analyses

FLH was subjected to anion exchange chromatography (IEX) on a MonoQ 5/5 column (GE Healthcare, Wisconsin, USA) and eluted with a linear NaCl gradient (0 to 0.7 M). The FLH subunit dissociation procedure was performed according to the method of Swerdlow et al. [16]. Briefly, purified FLH in buffer A (130 mM glycine-NaOH, pH 9.6, 10 mM EDTA) was loaded into a MonoQ HPLC column equilibrated with buffer A and eluted with a linear gradient of 0–70% buffer A containing 1.0 M NaCl. Chromatography was monitored at 280 nm, and elution fractions were collected and analyzed by SDS-PAGE [28].

### Humoral immune response determination

Two-month-old female BALB/c, C57BL/6 or C3H/HeJ mice were used. Three mice per group were immunized as follows. On day 1, the animals received 200 µg of FLH or the same amount of CCH or KLH as a control in 100 µl PBS intraperitoneally (ip), and the immunization was repeated on day 15. Ten days after, the mice were bled, and sera were obtained to determine the humoral immune response against each hemocyanin using an ELISA as previously described [29]. Briefly, 96-well polystyrene plates (Thermo Scientific) were incubated with 100 µl/well of a 10 µg/ml solution of each hemocyanin in PBS, and then were blocked with 1% PBS-casein. Serial 2-fold dilutions of the immune sera in blocking buffer were incubated for 1.5 h at 37°C. The plates were washed and 100 µl/well of goat anti-mouse IgG serum conjugated with alkaline phosphatase (ALP) (Thermo Scientific) diluted 1/2,500 in blocking buffer was then added to the wells and incubated at room temperature. The plates were then washed and developed by adding pNPP. The absorbance was measured at 405 nm.

To evaluate the carrier property of FLH, the hemocyanins were coupled to 2,4-dinitrofluorobenzene (DNFB) according to Sanger [30]. Briefly, 5 mg of each protein was dissolved in 1 M sodium bicarbonate. Then, 25 µl DNFB was added and mixed vigorously for 45 min at 37°C. Finally, the samples were dialyzed against PBS. Groups of mice were injected ip with 300 µg of the DNFB-conjugated hemocyanins in the presence of complete Freund's adjuvant (Pierce-Endogen) for the first immunization (day 1) and with incomplete Freund's adjuvant (Pierce-Endogen) for the second (day 18) immunization. Ten sera were obtained. The presence of specific IgG antibodies against DNFB was detected in the sera using an indirect ELISA as described above, although the plates were incubated with DNFB coupled to bovine sera albumin (BSA). Titers were defined as the reciprocal of the serum dilution with half of the maximum absorbance.

### Experimental immunotherapy schedule

Two-month-old C57BL/6 female mice were either primed with each hemocyanin or left untreated and then challenged with B16F10 melanoma cells using the method of Moltedo et al. with modifications [20]. In brief, 2 weeks prior to tumor implantation, groups of 7 mice were randomized and primed subcutaneously (sc) with 400 µg/100 µl PBS containing FLH, Ox-FLH, CCH or KLH independently or with PBS as the vehicle control. After day 20, the mice were challenged with an intralesional sc injection of 1.5×10^5^ melanoma cells in 100 µl DMEM into the right flank. Immunotherapy was administered for 6 consecutive days or 3 times every 7 days after challenge with the tumor cells. For the experiments using unprimed mice, a group of 5 mice was randomized and injected in the right flank with 1.5×10^5^ B16F10 cells, and 7, 14 and 21 days later, a subcutaneous injection with 1 mg FLH/100 µl PBS was administered intralesionally. The control mice received 100 µl PBS. Tumor dimensions were measured every 3 days, through day 25 (prior to the exponential growth of the tumor cells), and the tumor volume was calculated using the ellipsoid formula.

### Tumor infiltration analysis

Groups of 5 C57BL/6 mice were challenged via a sc injection of 1.5×10^5^ melanoma cells in 100 µl and were treated with FLH or PBS as described above. Fresh tumor-infiltrating lymphocytes, natural killer (NK) cells and macrophages were prepared from the tumors at day 18 of the bioassay, as described by Junankar et al. [31]. The cells were stained for specific cell surface markers using anti-CD4/CD3, anti-CD8/CD3, anti-CD19, anti-NK1.1 and anti-F4/80 antibodies (BD Biosciences Pharmingen, USA). The data were acquired on a FACScan flow cytometer (Becton-Dickinson, USA) and were analyzed using WinMDI and FlowJo software (Los Angeles, CA, USA). The results of the experiments are expressed as the percentages of tumor infiltrate-specific cells among the total immune cell population.

### Cytokine determination in mice sera and DC cultures

To measure the level of serum IFN-γ, the experimental and control mice were bled on day 13 of the bioassays. Supernatants for cytokine secretion were obtained from C57Bl/6 mouse bone marrow-derived DC cultures, as described by Lutz et al. [32]. Cells were seeded at 1.5×10^5^ cells/ml in 96-well plates and harvested after 24 h of incubation with each hemocyanin in different concentrations (100, 500 and 1,000 µg/ml) and assessed for TNF-α, IL-6, IL-12p40 and IL-23. The purity of the DCs was determined by flow cytometry and found superior to 80%. LPS (5–10 ng/ml) and PBS were used as positive and negative controls, respectively. These cytokines were measured using commercial ELISA kits according to the manufacturer's instructions (R&D systems, Minneapolis, MN, USA). As a control, a sample of the culture medium alone was included.

### Statistical analyses

The results of the experiments are expressed as the mean ± SE. Comparisons between groups were made using 1-way or 2-way ANOVAs and the Bonferroni post-test. The survival rate was estimated using the Kaplan-Meier method and the log-rank test. The analyses were performed using GraphPad Prism software (USA). For cytokine determination experiments, the unpaired Student's t-test was used.

## Results

### FLH consists of a single polypeptide chain

**Figure 1:**
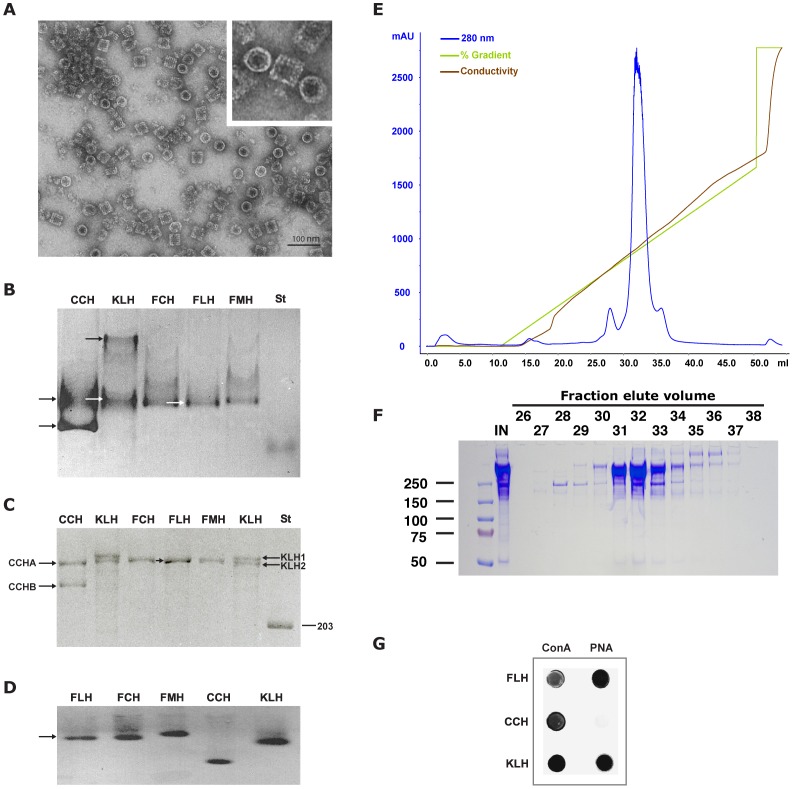
Biochemical characterization of FLH. (**A**). TEM of FLH negatively stained. The low-magnification micrograph of the purified protein shows the top (circles) and lateral (rectangles) views of the molecules. The insert shows a high-magnification view of the FLH molecules. (**B**). SDS-PAGE analysis under non-reducing conditions on a 3–7% polyacrylamide gradient gel stained with Coomassie blue. FLH shows one polypeptide in contrast to CCH and KLH that possess two (arrows). As controls, FCH and FMH were included. (**C**). SDS-PAGE analysis under reducing conditions on a 3–12% gradient polyacrylamide gel. The two CCH and KLH subunits are noted in contrast to the one subunit of FLH. (**D**). Native gel agarose electrophoresis. The image shows a single compacted band for each hemocyanin. (**E**). Chromatography of dissociated FLH. The previously dissociated protein was subjected to IEX HPLC on a MonoQ 5/5 column and eluted with a linear NaCl gradient (0 to 70%, green line). The chromatography was monitored at 280 nm (blue line). (**F**). SDS-PAGE analysis under reducing conditions of the elution fractions from the chromatography of E. The presence of a single main polypeptide in FLH was confirmed (**G**). FLH oligosaccharide pattern. Hemocyanins were blotted onto a nitrocellulose membrane, incubated with biotinylated lectins ConA and PNA and developed with avidin-FAL followed by NBT/BCIP detection.

Analysis of purified negatively stained FLH by TEM revealed a homogeneously sized protein with a quaternary structure that corresponded to the classical hollow, cylindrical didecamers of gastropods hemocyanins [21], [23] with an approximate diameter of 350 Å (Figure 1A). As shown in Figure 1B, the subunit composition of FLH in non-reducing conditions included a single polypeptide chain, similar to the closely related hemocyanins isolated from other *Fissurella* species, such as FCH and FMH; in contrast, KLH and CCH each possess 2 subunits. The molecular weight of the FLH subunit was approximately 350 kDa under reducing conditions (Figure 1C). Analysis using native agarose gels showed that the electrophoretic mobility of FLH was similar to that of KLH (Figure 1D). Additional IEX HPLC analyses with dissociated FLH demonstrated a single elution peak (Figure 1E), which was composed of a single polypeptide chain as determined by SDS-PAGE analysis. A second lower molecular weight band was observed, due to the dissociating condition, which induces some degradation of the protein (Figure 1F). Similar analyses of dissociated KLH and CCH revealed 2 peaks corresponding to 2 subunits [16], [23].

**Figure 2:**
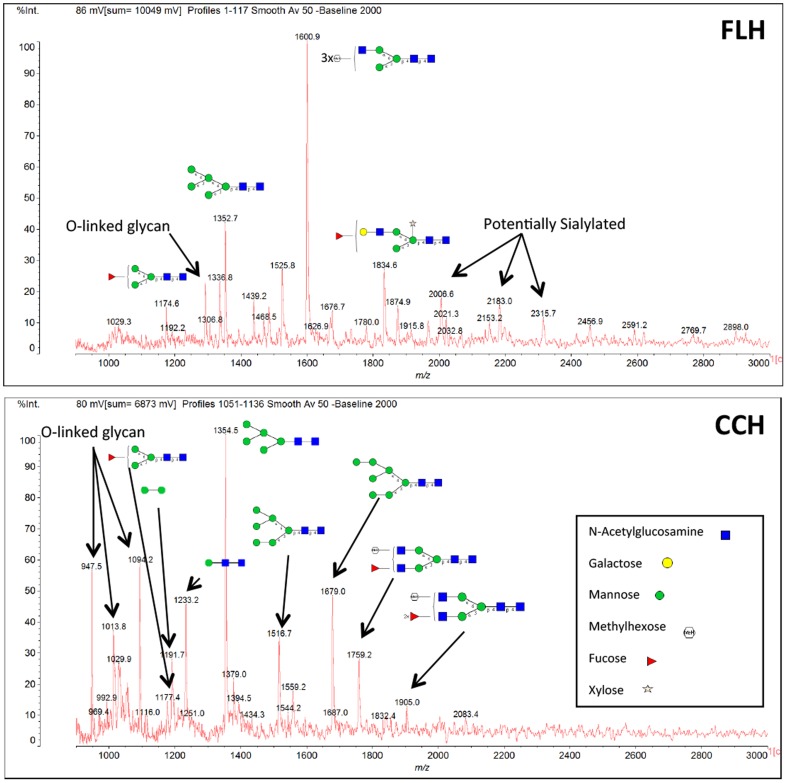
N- and O-linked oligosaccharide profile of FLH and CCH. The structure assignment was based on general knowledge of possible glycosylation in mollusk. The most likely structure was drawn on the spectra; however, additional monosaccharide matches were found for some masses.

The knowledge of the carbohydrate moieties present in mollusk hemocyanins has been essential for understanding their organization, antigenicity and biomedical properties [33]–[35]. Some hemocyanins possess sugar moieties, such as mannose-rich oligosaccharides and the Thomsen-Friedenreich antigen disaccharide (T antigen disaccharide Galβ1-3GalNAcα1-Ser/Thr), which may play roles in their immune-related properties [36], [37]. To analyze the general glycosylation pattern of FLH, qualitative dot blots were performed using ConA and PNA. The results indicated that FLH possessed both types of carbohydrate moieties, N-linked and O-linked, similar to KLH but different from CCH (Figure 1G). The mass spectrometric analysis of the FLH and CCH oligosaccharides showed the FLH seems to have more microheterogeneity in the N-linked oligosaccharides compared to CCH; the biggest difference between FLH and CCH is the presence of the xylose in the FLH, a sugar also reported in KLH [36], [37]. FLH also showed evidence of oligosaccharides containing sialic acid (although minor, at m/z 2007.6, 2316.8 and 2458.0) which CCH did not. Both CCH and FLH have high-mannose oligosaccharides, as well as fucosylated oligosaccharides (Figure 2); these sugar moieties also has been identified on KLH [36], [37].

Altogether, the biochemical data indicated that FLH is a unique hemocyanin composed of a single subunit. In addition, FLH has a similar carbohydrate composition to KLH but not to CCH, which may be explained by their phylogenetic relationship. *M. crenulata* and *F. latimarginata*, which produce KLH and FLH, respectively, are classified within the *Archaeogastropoda*: *Fissurellidae*, whereas CCH belongs to the *Neogastropoda*: *Muricidae*, a distant group of gastropod mollusks [38].

### Superior immunogenicity of FLH versus CCH and KLH

**Figure 3:**
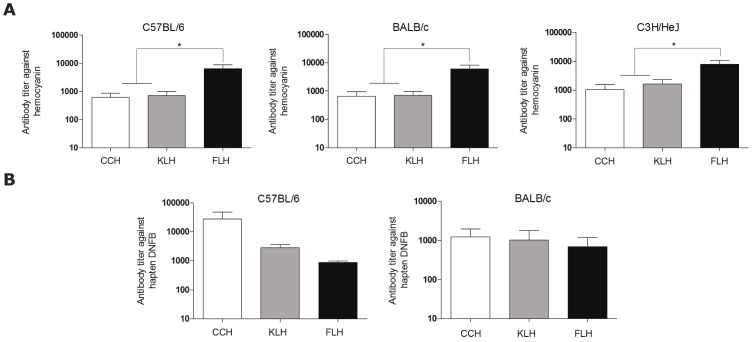
FLH is more immunogenic than CCH and KLH. (**A**). Humoral immune response against hemocyanins. Groups of 3 mice were immunized ip with 200 µg of FLH or CCH and KLH in PBS, and the immunization was repeated 15 days later. Ten days later, specific antibody titers were determined by ELISA from serially diluted sera. The mouse IgG antibodies were revealed using goat anti-mouse IgG ALP-conjugated serum; subsequently, pNPP was added. The reaction was read at 405 nm. Data are shown as mean + SEM of *n* = 3 mice per group and are representative of two independent experiments, *p<0.05 between FLH *versus* KLH and CCH. (**B**). Determination of the carrier property of FLH. The hemocyanins were coupled to DNFB; subsequently, groups of 3 C57BL/6 or BALB/c mice were immunized ip with 300 µg of each conjugate with Freund's adjuvants as a repository. Ten days after, the titers against DNFB were determined by ELISA. FLH *versus* CCH and KLH p not significant.

We first evaluated the immunogenicity of FLH and compared it to that of CCH and KLH. For this purpose, mice of different strains (C57BL/6, BALB/c and C3H/HeJ) were injected ip on days 1 and 14 with 200 µg of each hemocyanin without adjuvants. Specific antibody titers were determined by ELISA 10 days after the second immunization. The results showed that FLH produced a significantly higher antibody titer (approximately 1/10,000) than CCH and KLH (approximately 1/1,000) in all of the strains used (Figure 3A). Next, to assess the potential of FLH as a carrier, DNFB was coupled to the hemocyanins in a hapten model. C57BL/6 and BALB/c mice were immunized with 300 µg of each conjugate, with a similar schedule as described above, although CFA and IFA were used in the primary and secondary immunization procedures, respectively. As shown in Figure 3B, the levels of anti-DNFB antibodies were higher in the C57BL/6 mice immunized with CCH-DNFB (approximately 1/10,000) than in the mice immunized with FLH-DNFB and KLH-DNFB (approximately 1/1,000). In contrast, in BALB/c mice, similar titers against DNFB (approximately 1/1,000) were obtained when using all of the hemocyanins as carriers. The titer differences between the mouse strains may be explained by some type of genetic control of the immune response against hemocyanins [39], [40]. In addition, C57BL/6 and BALB/c mice are known to be prone to develop a Th1 and Th2-type activation, respectively. Taken together, these results indicate that FLH generates a more robust specific humoral immune response than CCH and KLH without adjuvant; however, its performance as a carrier for haptens mixed with CFA was similar to that of KLH.

### FLH induces a significant anti-tumor effect in the B16F10 murine melanoma model

**Figure 4:**
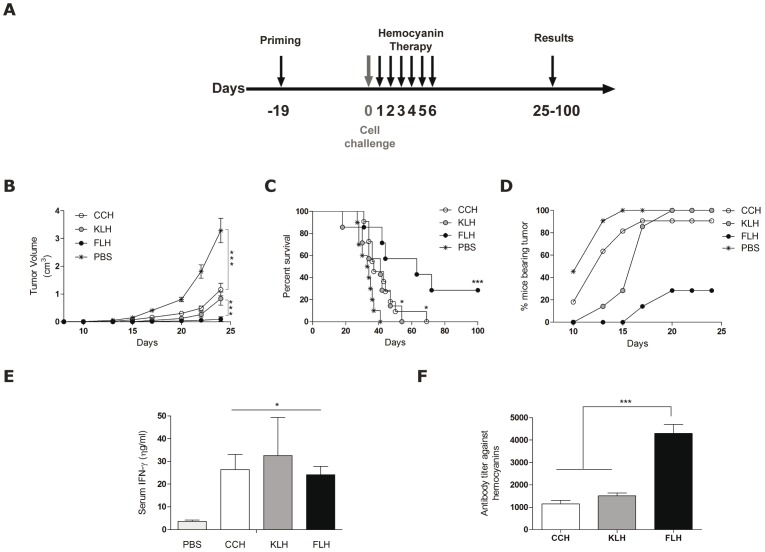
FLH possesses greater anti-tumor activity than CCH and KLH in the B16F10 mouse melanoma model. (**A**). The schedule of the bioassay. Groups of 5–7 mice were sc primed with FLH, CCH or KLH (400 µg/100 µl PBS). After 19 days, the mice were challenged sc with 1.5×10^5^ B16F10 melanoma cells, and they then received intralesional injections with 100 µg of each hemocyanin for 6 consecutive days. Data are representative of two independent experiments. (**B**). Effect of FLH on melanoma growth. The tumor volume was measured every 3 days; ***p<0.001 between CCH, KLH and FLH *versus* PBS and ***p<0.001 between FLH *versus* CCH and KLH. (**C**). Animal survival. Kaplan-Meier survival analysis were compared using the log-rank test; ***p<0.001 FLH *versus* PBS, *p<0.05 between CCH and KLH *versus* PBS and *p<0.05 between CCH and KLH *versus* FLH. (**D**). Effect of FLH on melanoma growth. Tumor progression was measured for 25 days following the different treatments. (**E**). Levels of IFN-γ in the sera of mice. The levels of IFN-γ in mouse sera were determined by ELISA on day 13 of the bioassay; *p<0.05 hemocyanins *versus* the control with PBS. (**F**). Humoral immune response against hemocyanins. Anti-hemocyanin antibody titers in mouse sera were determined by ELISA on day 13 of the bioassay; ***p<0.001 FLH *versus* CCH and KLH.

Hemocyanins are well-known immunotherapeutic molecules used in the treatment of superficial bladder cancer; however, these molecules have not been widely investigated for use in other types of cancer. KLH was shown to inhibit the appearance of tumors until 16 days after melanoma HTB68 cell implantation [41]. Furthermore, we showed that both CCH and KLH demonstrated anti-tumor effects in the B16F10 murine melanoma model [25]. Because FLH induced a more robust antibody response when compared to KLH and CCH, it was important to determine whether this property would also translate to improved anti-tumor effects in this melanoma model. Mice previously immunized with FLH, CCH or KLH were challenged with B16F10 melanoma cells and underwent hemocyanin immunotherapy (400 µg/dose) over a period of 6 consecutive days (Figure 4A). Mice treated with FLH demonstrated reduced tumor growth rates, and the percentages of mice bearing tumors decreased significantly in comparison to mice that were treated with CCH and KLH (Figure 4B, 4C and 4D). Additionally, the mouse survival analysis, which was continued until day 100 of the bioassay, indicated that each mouse that received KLH and CCH therapy died by day 70, in contrast to only 70% of the mice subjected to FLH therapy; furthermore, the remaining mice in the FLH group survived more than 100 days after tumor challenge (Figure 4C).

To further investigate the differences observed in the potent effect of FLH and the possible mediators involved, we evaluated the production of IFN-γ and the secretion of anti-hemocyanin antibodies in serum collected on day 13 of the bioassay. No significant differences were observed in the secretion of IFN-γ between hemocyanin treatments, indicating that other differences between the immune response to hemocyanins are involved in the Th1 polarization induced by these proteins (Figure 4E). In addition, the mice that received FLH therapy demonstrated significantly higher antibody titers than those receiving CCH and KLH therapies, which confirmed our previous results and supported the potent immunogenicity of FLH (Figure 4F).

**Figure 5:**
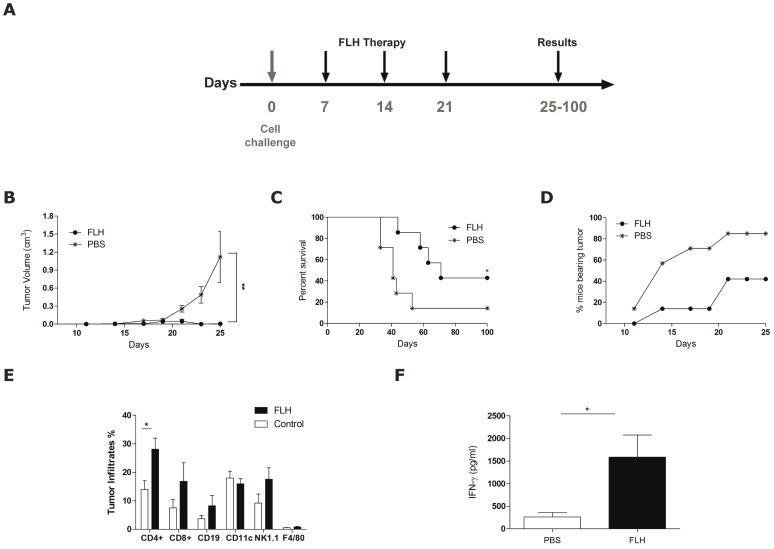
Immunotherapeutic effect of FLH in the B16F10 mouse melanoma model does not require priming. (**A**). Schedule of the bioassay. (**B**). Effect of FLH on melanoma growth. Tumor growth was followed every 3 days, and each group contained 7 mice; ***p<0.001 between FLH and PBS. (**C**). Effect of FLH on the survival of mice. The log-rank test was used to compare the curves; *p<0.05 between FLH and PBS. (**D**). Kinetics of tumor appearance in mice. The percentages of mice bearing tumors were determined visually and by palpation. (**E**). Tumor-infiltrating immune cells. The quantitative analysis of tumor infiltration by CD8/CD3+, CD11c+, NK1.1+, F4/80+, CD4/CD3+ and CD19+-positive cells in single-cell suspensions derived from B16F10 tumors was performed on day 18 of the bioassay. The results are expressed as the percentages of total viable immune cells. Each group consisted of 3–4 mice; *p<0.05. (**F**). IFN-γ measurements from mouse sera were obtained on day 18 of the bioassay (same mice and day as the experiment shown in E); *p<0.05 FLH *versus* PBS.

The priming requirements for the anti-tumor effects of KLH and CCH in superficial bladder cancer have been established [20], [42], and we therefore asked whether priming with FLH prior to challenge with melanoma cells would be required for its anti-tumor effects. In therapeutic experiments, mice were challenged with B16F10 cells and after one week the immunotherapy was initiated, with 1 mg/dose of FLH locally at the tumor site on days 7, 14 and 21 post-challenge (Figure 5A). The mice treated with FLH demonstrated a significant delay in tumor growth rate (Figure 5B), fewer of the mice bore tumors (Figure 5D), and 40% of the mice survived up to day 100 of the experiment (Figure 5C). In addition, mice bearing tumors were euthanized on day 18, and the tumor-infiltrating cells were isolated and analyzed by flow cytometry. The data revealed a significant increase in tumor-infiltrating CD4^+^ lymphocytes, a trend that was similar for NK, CD19 and CD8^+^ T cells (Figure 5E). As expected, the sera of these mice also contained increased levels of IFN-γ in comparison to the control (Figure 5F). Altogether, these results demonstrated effective anti-tumor activity of FLH in the B16F10 melanoma model.

### Deglycosylated FLH demonstrates an attenuated anti-tumor effect

**Figure 6:**
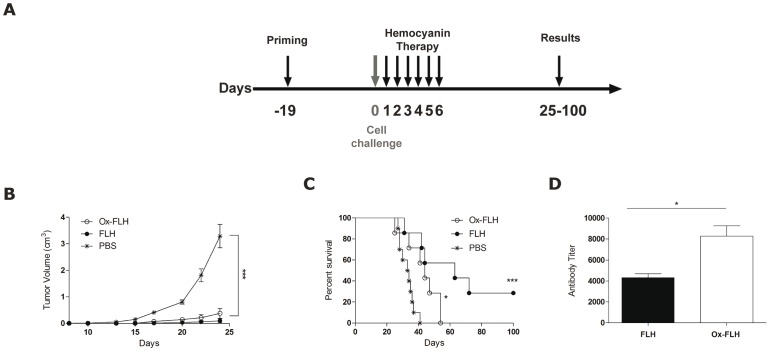
Effect of periodate treatment on anti-tumor properties of FLH in the B16F10 mouse melanoma model. (**A**). Schedule of the bioassay. Groups of 5–7 mice were sc primed with FLH or Ox-FLH (400 µg/100 µl PBS). After 19 days, the animals were challenged sc with 1.5×10^5^ B16F10 melanoma cells, and they then received intralesional injections of 100 µg of each hemocyanin for 6 consecutive days. (**B**). Effect of Ox-FLH on melanoma growth. Tumor growth was followed every 3 days until day 25, and each group consisted of 5 mice; ***p<0.001 between FLH and Ox-FLH *versus* PBS. (**C**). Effect of Ox-FLH on the survival of mice. Mouse survival was recorded until day 100 of the bioassay. The survival curves were compared using the log-rank test; ***p<0.001 FLH *versus* PBS, *p<0.05 between Ox-FLH *versus* PBS. (**D**). Ox-FLH was more immunogenic than its native counterpart; *p<0.05 between FLH and Ox-FLH.

Previous authors have suggested that the anti-tumor effects of hemocyanins may be mediated by their glycosylation [14], [37]. Therefore, a deglycosylated form of FLH (Ox-FLH) was produced to evaluate the role of FLH oligosaccharides in tumor immunotherapy. Two groups of mice were immunized in parallel with FLH and Ox-FLH in the same schedule shown in Figure 4 (Figure 6A). The results demonstrated that Ox-FLH treatment led to a similar delay in tumor growth as native FLH in comparison to the PBS control until day 25 (Figure 6B). However, the survival rates of mice treated with native FLH were significantly improved compared to those of the Ox-FLH and PBS groups until day 100 (Figure 6C). These results suggest that the anti-tumor effect of Ox-FLH is less durable, and that oligosaccharides and/or structural features affected by the chemical treatment of FLH, are fundamental for its anti-tumor effects. However, chemical treatment of FLH did not alter antibody production in challenged mice, and Ox-FLH treatment led to a slightly higher antibody titer compared to FLH (Figure 6D). It is known that the fixation of proteins enhances their resistance to lysosomal proteolysis and therefore increases their immunogenicity [43]. Of note, we did not detect evident allergic reactions or toxic effects in mice treated with FLH or Ox-FLH throughout our studies. In addition, the cytotoxic effect of Ox-FLH on B16F10 melanoma cells cultured in vitro was determined by the MTT assay and we did not found a cytotoxic effect.

### Deglycosylation of FLH abolishes robust DC maturation

**Figure 7:**
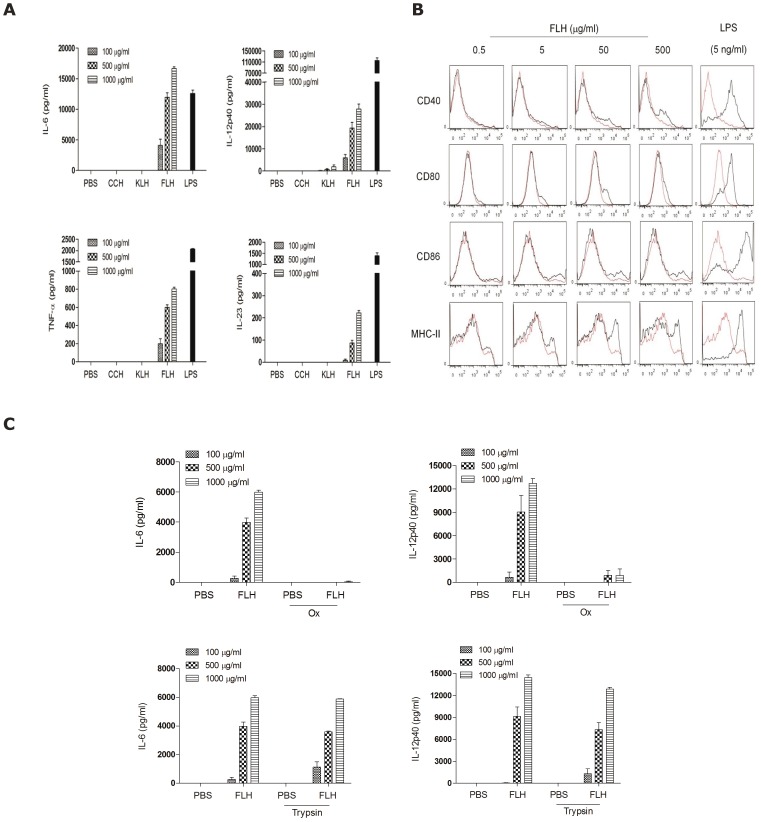
FLH induces pro-inflammatory cytokines more rapidly than CCH and KLH in DCs, and Ox-FLH abolishes this secretion. (**A**). FLH rapidly induced the dose-dependent secretion of pro-inflammatory cytokines. DCs from C57BL/6 mouse strain were cultured with FLH, CCH or KLH (100, 500 or 1,000 µg/ml) for 24 h, and the supernatants were collected to determine the secretion levels of IL-6, TNF-α, IL-12p40 and IL-23 using commercial ELISA kits. PBS was used as the negative control. Data are shown as mean + SEM of three independent experiments. (**B**). The FLH-induced, reduced up-regulation of DC maturation markers. DCs were cultured in similar conditions as in (A) and were analyzed for maturation markers (CD80, CD86, CD40 and MHC-II) by flow cytometry. (**C**). Effect of trypsin-digested Ox-FLH and FLH on the secretion levels of pro-inflammatory cytokines. The DCs were cultured for 24 h with Ox-FLH, native FLH or trypsin-treated FLH. The supernatants were obtained and analyzed for IL-6 and IL-12p40 using commercial ELISA kits. Data are shown as mean + SEM of two independent experiments, *p<0.p<0.05 = *, p<0.01 = ** and p<0.001 = ***, unpaired Student's t-tests.

Because DCs play a pivotal role in the regulation of immune responses and tumor immunosurveillance [44], [45], we next investigated the effects of FLH on murine DCs cultured *in vitro*. In contrast to KLH and CCH, we observed that FLH induced the dose-dependent secretion of several pro-inflammatory cytokines, including IL-6, TNF-α, IL-12p40 and IL-23 (Figure 7A). In addition, FLH induced low upregulation of co-stimulatory molecules (CD80, CD86, CD40) and MHC-II in comparison to LPS, which was used as a positive control (Figure 7B).

Because chemical deglycosylation of FLH impaired its anti-tumor effect, we evaluated Ox-FLH for its capacity to elicit DC activation. DCs were incubated for 24 h with Ox-FLH or native FLH, and the secretion of IL-6 and IL-12p40 was analyzed. The results showed that the secreted levels of both cytokines were practically abolished when DCs were treated with Ox-FLH (Figure 7C). Furthermore, supporting the fact that glycosylations are crucial for FLH-induced DC maturation, we proceeded to partially digest the protein with trypsin, which generates smaller fragments and disrupts the cylindrical quaternary structure of the protein while leaving glycosylations intact. As shown in Figure 7C, small fragments of FLH maintained their non-specific immunostimulatory effects, and DC maturation was observed in a dose-dependent manner.

## Discussion

This study introduced FLH as a novel immunomodulatory hemocyanin from *Fissurella latimarginata*. FLH demonstrated greater immunostimulatory properties than traditional KLH and CCH, inducing effective anti-tumor immune responses in a melanoma model. We selected the B16F10 melanoma model because the effectiveness of immune therapies in this model may be considered a reasonable predictor of the effectiveness of immune therapies against human tumors [46]. In addition, KLH is currently used to increase the immunogenicity of some specific glycolipid and mucin-like tumor associated antigens to eliminate the recurrence of such tumors [7].

The data shown here indicate that the mechanism underlying the anti-tumor effect of FLH is, at least in part, mediated by glycosylation. Indeed, it has been shown that KLH contains a variety of unique N-glycans with Gal(*β*1-6)Man- structural determinants [37]. As mentioned above, the presence of cross-reacting epitopes, such as the T-antigen determinant in the bladder and other epithelial cancer cells, could improve the efficacy of KLH as an immunotherapeutic agent for the treatment of superficial bladder cancer [36]. The results showed that while CCH, KLH and FLH exposed mannose-rich oligosaccharides, only KLH and FLH exposed the T-antigen determinant. However, our results showed that KLH and FLH had different anti-tumor behaviors, indicating that the expression of the T-antigen alone does not explain the effects observed with FLH. It is possible that the arrangement of the GalNAc residues between FLH and KLH may be different and more exposed in FLH, allowing their interaction with some APC receptors to stimulate the innate immune response. Using surface plasmon resonance, it has also been shown that differences in the arrangements, but not the quantities, of GalNAc residues on mucin repeats affect their association rate constants with lectin [47].

Studies have shown that the anti-tumor effect of hemocyanins requires previous priming [20], [42], [48]. However, we observed that FLH, unlike CCH and KLH, had potent anti-tumor activity on murine melanoma without previous priming. To explain this effect, we hypothesized that FLH may induce a more potent inflammatory milieu than CCH and KLH. To test this hypothesis, we studied the effects of these hemocyanins on the expression of cytokines and maturation markers by DCs. In contrast to KLH and CCH, FLH activated the rapid production of pro-inflammatory cytokines, such as IL-6, TNF-α and especially high levels of IL-12p40, which play pivotal roles in coordinating innate and adaptive immunity toward a Th1 response [49]. In humans, direct maturation of DC activity has been demonstrated in response to the mannosylated carbohydrates present in KLH [13]. In this respect, we observed that secretion of IL-12p40 and IL-6 by DCs was dependent on the integrity of the glycosylation of FLH, suggesting that these carbohydrates play a crucial role in innate immune recognition of this protein. The mechanisms associated with these effects are still poorly understood; however, it is likely that, besides the mannose receptor, another C-type lectin receptor expressed by DCs may be involved [50]. In support of this idea, some C-type lectin receptors on the surfaces of antigen presenting cells have been associated with potent anti-tumor responses characterized by the secretion of Th1 cytokines and the generation of cytotoxic T-lymphocytes [51]. Interestingly, FLH induced only low up-regulation of DC maturation markers, in contrast to LPS. In this context, we have previously shown that CCH is slowly processed by murine DCs cultured *in vitro* and neither the up-regulation of MHC molecules nor the up-regulation of costimulatory molecules were observed [25].

We established that FLH is an invariable protein because it is formed by a unique subunit, unlike KLH. This feature of FLH may be advantageous for the development of new experimental vaccines that use hemocyanins as carriers of tumor associated antigens. The main disadvantages of KLH as a carrier for these antigens in human trials relate to its heterogeneity [52], the variability in its proportion of subunits [16] and the low induction of long-lasting titers of IgM- and IgG-specific antibodies [53], [54]. In this respect, the evaluation of FLH as a carrier for the DNFB has demonstrated similar results to those observed with KLH, and FLH was not superior to CCH. This result may be due to the biochemical differences in the residues exposed by different hemocyanins when chemically coupled to DNFB. In support of this idea we previously reported spectral differences between CCH and KLH [55].

Altogether, these results led to speculation as to the intrinsic structural features responsible for the immunogenicity and anti-tumor effects of FLH. We hypothesized that one peculiar characteristic of FLH may be related to its oligosaccharide components, which may act as natural immunostimulants via their interaction with a specific or putative receptor located on the surface of DCs. FLH binding to this receptor may induce IL-12 secretion and the subsequent activation of other innate immune cells, such as NK cells, which then secrete IFN-γ. Subsequently, FLH localized to phagosomes is slowly processed into peptides by DCs, and these xenogenic peptide sequences from FLH may stimulate CD4^+^ lymphocytes more strongly than the peptide sequences from other hemocyanins, resulting in increased levels of inflammatory cytokines in the regional lymphoid ganglions. Furthermore, this environment could lead to the proliferation of anti-tumor-associated CD8^+^ lymphocytes through the indirect stimulation of latent specific responses (i.e., the bystander effect), which could break tolerance towards the tumor. This hypothesis is supported by several lines of evidence. First, DCs express a wide repertoire of saccharide-recognizing surface receptors [56]; of these, the mannose receptor is likely partially involved in this process [13], [25]. Second, hemocyanins are slowly processed [25], and third, hemocyanins stimulate T-helper cell responses, supporting the secretion of cytokines such as IFN-γ and IL-2 by tumor reactive T cells localized in the regional lymph nodes [11]. This inflammatory environment enhances NK cell activity in patients with superficial bladder cancer [57] and in mouse models of this cancer [20], and it is known that NK cells are strongly stimulated by IL-2 to differentiate into lymphokine-activated killer cells [58]. Finally, NK cells can delay tumor growth by means of antibody-dependent cell-mediated cytotoxicity of effector cells [59]. In this respect, the anti-tumor activity of KLH has been associated with antibody cross-reactivity between cell-surface tumor antigens and KLH oligosaccharides, which may promote this type of cytotoxicity [60].

In conclusion, the presented results lead us to hypothesize that hemocyanins from the gastropod *Fissurella latimarginata*, designed as FLH, can induce an inflammatory milieu more rapidly than CCH and KLH, resulting in a potent innate immune response and an enhanced anti-tumor adaptive immune response against melanoma cells. However, in order to generalize the findings, additional tumor models will be necessary. Consequently, FLH is a potential new marine adjuvant for immunization and possible cancer immunotherapy. Finally, a detailed molecular understanding of the receptors and signaling pathways involved in hemocyanin-induced immunostimulatory processes will be required to elucidate the basis for the qualitative and quantitative differences between these interesting and useful proteins.
